# Antiaging effects of dietary supplements and natural products

**DOI:** 10.3389/fphar.2023.1192714

**Published:** 2023-06-27

**Authors:** Lulu Gao, Xudong Liu, Xiaoyan Luo, Xiaofan Lou, Pusen Li, Xian Li, Xiaomeng Liu

**Affiliations:** School of Public Health, Xinxiang Medical University, Xinxiang, Henan, China

**Keywords:** dietary supplements, natural products, aging, aging-realated disease, antiaging molecules

## Abstract

Aging is an inevitable process influenced by genetics, lifestyles, and environments. With the rapid social and economic development in recent decades, the proportion of the elderly has increased rapidly worldwide, and many aging-related diseases have shown an upward trend, including nervous system diseases, cardiovascular diseases, metabolic diseases, and cancer. The rising burden of aging-related diseases has become an urgent global health challenge and requires immediate attention and solutions. Natural products have been used for a long time to treat various human diseases. The primary cellular pathways that mediate the longevity-extending effects of natural products involve nutrient-sensing pathways. Among them, the sirtuin, AMP-activated protein kinase, mammalian target of rapamycin, p53, and insulin/insulin-like growth factor-1 signaling pathways are most widely studied. Several studies have reviewed the effects of individual natural compounds on aging and aging-related diseases along with the underlying mechanisms. Natural products from food sources, such as polyphenols, saponins, alkaloids, and polysaccharides, are classified as antiaging compounds that promote health and prolong life *via* various mechanisms. In this article, we have reviewed several recently identified natural products with potential antiaging properties and have highlighted their cellular and molecular mechanisms. The discovery and use of dietary supplements and natural products that can prevent and treat multiple aging-related diseases in humans will be beneficial. Thus, this review provides theoretical background for existing dietary supplements and natural products as potential antiaging agents.

## 1 Introduction

**FIGURE 1:**
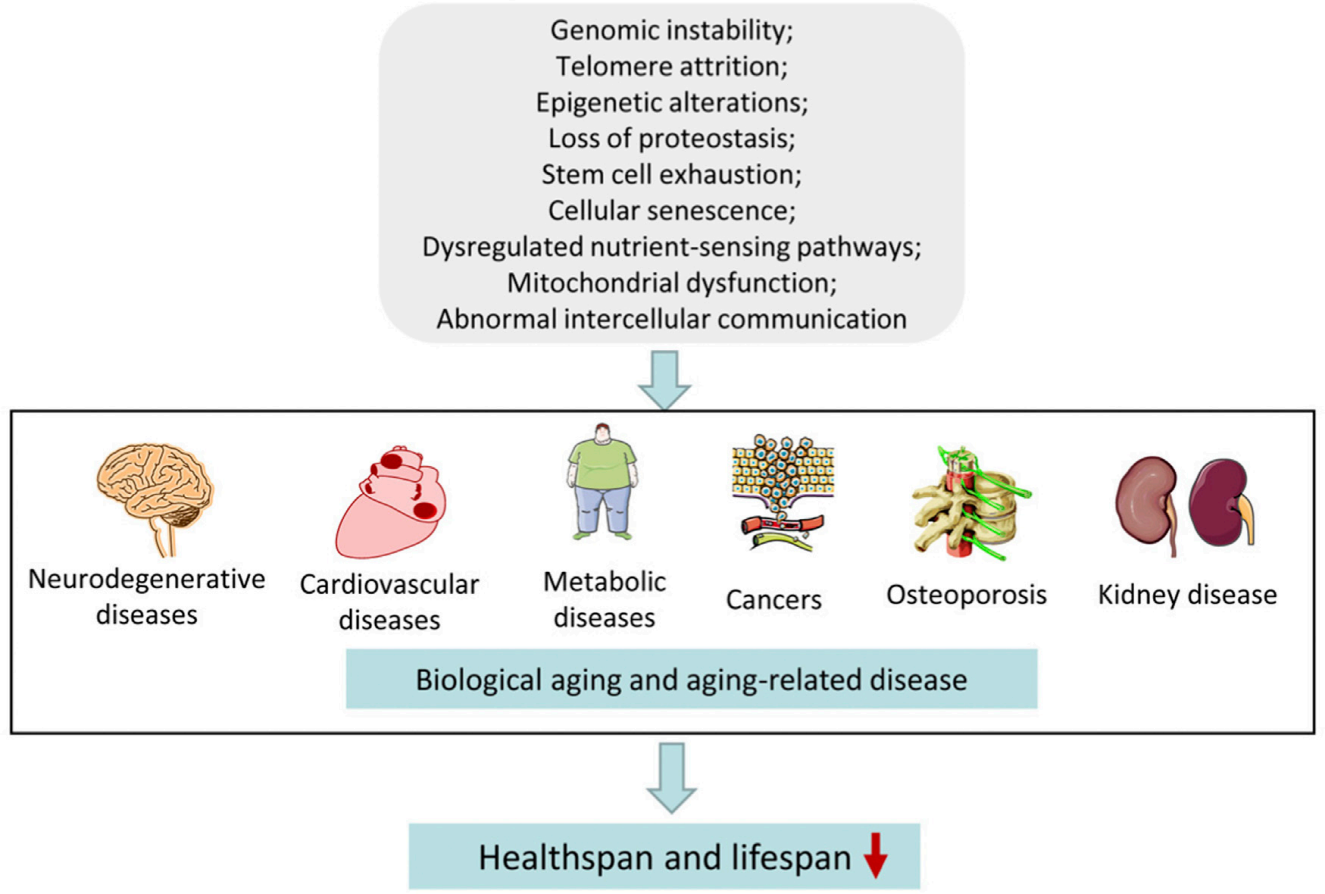
Hallmarks of aging and aging-related diseases.

The physiological and psychological adaptability of organisms to the environment diminishes with age, leading to functional decline and increased risks of disease and death (Rahman et al., 2021). The aging process can be divided into two categories: physiological and pathological aging. Physiological aging refers to the physiological degeneration occurring after maturity, while pathological aging involves age-related changes caused by various external factors such as various diseases. Since these two types are practically difficult to distinguish, aging can be referred to as a biological-psychological process during the final stages of individual growth and development, which is the inevitable result of a combination of many pathological, physiological, and psychological processes. The proportion of the elderly has tremendously increased worldwide in recent decades with rapid socio-economic development. In 2019, there were 1 billion people aged 60 years and older. It is estimated that this number will increase to 1.4 billion by 2030, and to 2.1 billion by 2050. The proportion of the elderly (aged >60 years) is estimated to rise to 22% of the world population by 2050 (Larbi et al., 2013). The rate of increase in the aging population is unprecedented and is expected to accelerate in the coming decades, particularly in developing countries (United Nations Department of Economic and Social Affairs, 2019; WHO, 2019). The aging process in different individuals may be driven by range of factors, including genomic instability, telomere attrition, epigenetic alterations, proteostasis loss, mitochondrial dysfunction, dysregulated nutrient-sensing pathways, cellular senescence, stem-cell exhaustion, and abnormal intercellular communication (Chuang et al., 2014). The classic hallmarks of molecular aging and aging-related diseases are shown in Figure 1. The increasing aging population is challenging in different ways for societies across the globe. Therefore, aging-related research is being focused on along with age-associated diseases. This includes exploring molecular mechanisms related to longevity and investigating safe and effective interventions for aging, which can systematically reveal the intrinsic mechanisms underlying aging and the influence of external environments on aging, promoting healthy aging.

As the quality of life continues to improve, more research is being focused on antiaging methods that are beneficial to physical and mental development, and on maintaining self-generated physical and mental health while extending life expectancy. Aging and longevity are regulated by various factors including lifestyle, nutrition, genetics, and exercise (Hosseini et al., 2021). Caloric restriction has been reported to delay age-related organ diseases and extend the lifespans of several species. Growing evidence reveals that certain phytochemicals have potential antiaging properties (Si and Liu, 2014). Natural products are defined as specific constituents or their metabolites in animals, plant extracts, insects, marine organisms, and microorganisms, as well as many endogenous chemical substances in humans and animals (Akter et al., 2021). Natural products for medicine and health have been enormously important and have been used for thousands of years to ameliorate disease symptoms or completely treat various disorders. Approximately 60% of existing drugs have been directly or indirectly derived from natural products (Ji et al., 2009; Akter et al., 2021). Natural products with various pharmacological properties also contribute to novel drug discovery and development (Mathur and Hoskins, 2017). Most studies have evaluated the antiaging effects of natural products in terms of the cardiovascular system, neurodegenerative diseases, metabolic diseases, cancers, and osteoporosis (Youssef et al., 2022). Natural products derived from food sources, such as polyphenols, saponins, alkaloids, and polysaccharides, have been recognized as antiaging molecules that can promote health and extend lifespan through multiple mechanisms (Chen Y. et al., 2022). This review focuses on examining the mechanisms underlying the antiaging effects and therapeutic properties of various natural products.

## 2 Active ingredients of natural products with antiaging properties

### 2.1 Polyphenols

Polyphenols are secondary metabolites commonly found in a variety of foods, which have potent antioxidant properties and can protect against various pathogens (Luo et al., 2021). Polyphenols possess antioxidant, anti-inflammatory, and anticancer properties, which contribute to the prevention of aging-related diseases such as cancers, nephrosclerosis, arthritis, and neurodegenerative and cardiovascular diseases (Arora et al., 2020). Polyphenols are classified into different types based on the number of phenolic rings. The principal classes are phenolic acids, flavonoids, stilbenes, and lignans. Phenolic acids can be subdivided into two groups based on their derivatives: benzoic acid derivatives (e.g., gallic acid and hydroxybenzoic acid) and cinnamic acid derivatives (e.g., ferulic acid, caffeic acid, and quinic acid). They are primarily found in fruits, vegetables, grains, coffee, and tea. Flavonoids have two aromatic rings connected by the three central carbon atoms. They are divided into several classes based on their structures, including flavones and flavonols (quercetin, rutin, and baicalein), flavanones and flavanonols (catechins in tea polyphenols and epigallocatechin gallate), isoflavones and isoflavanones (daidzein, genistein, and puerarin), anthocyanidins (geranium and delphinidin). The primary flavonoid sources are green tea, colored fruits and vegetables, soybeans, and chocolates. Stilbenes are polyphenols present in low quantities in the diet, including resveratrol, pterostilbene, and piceatannol. They are commonly found in various plants. Resveratrol is the most studied natural product with antiaging effects and has been reported to have anticancer effects in medicinal plant screening. However, due to the low quantities of stilbenes in the diet, they are unlikely to exhibit protective effects at normal nutritional intakes. Lignans consist of two phenylpropanes primarily derived from oleaginous seeds such as linseed, followed by algae, cereals, certain vegetables and fruits, and leguminous plants such as lentils. Several recent studies have focused on the effects of different types of polyphenols on healthy aging and/or aging-related diseases. Collectively, polyphenols have potent antioxidant, anti-inflammatory, and anticancer properties, which make them potentially valuable in preventing aging and aging-related diseases. The natural polyphenols and aging-related signaling pathways are provided in Table 1.

**TABLE 1:** Natural polyphenols and aging-related signaling pathways.

Natural product	Antiaging mechanisms	Outcomes	Resources	References
Quercetin	Anti-inflammatory, antioxidant, antimicrobial properties	↓ TNF-α, CRP, IL-6, IL-1β	Apples, berries, tomatoes, onions, lettuce	Shan et al. (2009), Kobori et al. (2015), Elbe et al. (2016), Diniz et al. (2020), Sugawara and Sakamoto (2020)
	Inhibits the IIS and MAPK, TGF-β1/Smad2/3, NF-κB, and Wnt/β-Catenin signaling pathways; activates the Nrf2 pathway	↓ iNOS, IL-12		
		↑ GSH/oxidized GSH		
		↓ D-lactate, diamine oxidase		
Luteolin	Anti-inflammatory, antioxidative, anticancer properties	↓ synthesis of both COX-2 and PGE2	Reseda luteola, parsley, celery, broccoli, rosemary, cardamom, anise, dandelion	Jia et al. (2015), Kim et al. (2017), Akter et al. (2021), Mu et al. (2021)
	Inhibits the SIRT3/ROS/MAPK, IΚBα/NF-κB, and JAK/STAT signaling pathways; improves mitochondrial dysfunction;	↓ proinflammatory cytokines (IL-6, TNF-α and IL-20)		
	inhibits endoplasmic reticulum stress;			
	SIRT6 agonist			
Catechins	Antioxidative, scavenging of free radicals, and antiapoptotic properties	↓ NADPH quinone, GSH, ACC1, FAS,	Tea, apples, persimmons, cacaos, grapes, berries	Wu et al. (2006), Li et al. (2008), Ye et al. (2012), Niu et al. (2013), Fei et al. (2017), Yi et al. (2017), Yuan et al. (2020)
	Activates the Nrf2/HO1, PI3K/AKT, SIRT1/PGC-1α, ketogenesis/SIRT3, and ERK signaling pathways; inhibits the NF-κB signaling pathway	↑ SOD scavenging		
Resveratrol	Regulates apoptosis, inhibits inflammation, suppresses oxidative stress, regulates gut microbiome; inhibits the TLR2/MyD88/NF-κB, and AKT/mTOR signaling pathways;	↑ PGC1α, UCP1, PRDM16, Cidea	Grapes, peanuts, pomegranates, blueberries, cocoa, red wine	Tomé-Carneiro et al. (2012), Yu and Li (2012), Wang et al. (2015), Moussa et al. (2017), Sung et al. (2017), Kim et al. (2018), Khan et al. (2019b)
	activates the Sirt1, Nrf2, and AMPK signaling pathways;	↑ HO-1, NQO-1, IL-10, SOD, CAT		
	improves mitochondrial function	↓ IL-1β, TNF-α, IL-8, ROS		
Curcumin	Induces autophagy, inhibits inflammation and oxidation, and maintains mitochondrial function	↓ ROS MDA GPX RNS	Curcuma longa	Zhu et al. (2014), Qin et al. (2018), Porro et al. (2019), Benameur et al. (2021), Meng et al. (2021), Cianciulli et al. (2022)
	Activates the AMPK, SIRT1, and Nrf2/Keap1 signaling pathways; inhibits the TLR4/MyD88/NF-κB, mTOR, and JAK/STAT/SOCS signaling pathways	↑ SOD, PPARγ activity		
		↓ MCP-1, COX-2, lipoxygenase, iNOS, MAPK		
Lignans	Anti-inflammatory and antioxidative properties. Inhibits the NF-κB, JNK/p38, and MAPK signaling pathways; activates the AMPK, PI3K/Akt, and Nrf2/HO-1 signaling pathways	↓ NO, ROS, iROS	Oilseeds, whole grains, legumes, fruits, vegetables, nuts, tea, coffee	(Jang et al., 2019) (Liu et al., 2021)

↓ decrease/reduce; ↑ increase/promote; IIS, insulin/insulin-like signaling; Nrf2, nuclear factor E2-related factor 2; UCP1, uncoupling protein 1; MAPK, mitogen-activated protein kinase; PGC1α, peroxisome proliferator-activated receptor-γ coactivator 1α; ROS, reactive oxygen species; GST, glutathione s-transferase; HO-1, heme oxygenase 1; NQO-1, NAD(P) H:quinone oxidoreductase 1; SIRT, sirtuin; PI3K, phosphatidylinositol-3-kinase; JNK, c-Jun N-terminal kinase; PRDM16, PR domain-containing 16; Cidea, Cell death-inducing DFFA-like effector A.

#### 2.1.1 Quercetin

Quercetin is the most widely studied flavonoid, primarily derived from apples, berries, tomatoes, onions, and lettuce. The hydrophilic glycoside (quercetin glucosylation) is the primary form of quercetin in plants that are not absorbed by intestinal cells directly. In intestinal epithelial cells, quercetin glycosides undergo various changes and are a part of crucial enzymatic reactions, such as hydrolysis, methylation, and sulfonylation by specific transferases and glucuronidation. Therefore, quercetin glycosides are absorbed and used by intestinal epithelial cells. Quercetin is metabolized in the intestine, and its methylated metabolite isorhamnetin is the major form found in serum (Shi et al., 2019). Quercetin exhibits anti-inflammatory, antioxidant, and antimicrobial properties and is used as a dietary supplement at a safe dose of 1 g/day with approximately 60% absorption (Yang et al., 2020; Deepika and Maurya, 2022).

In 1994, Hanasaki et al. (1994) reported that quercetin had the most robust rate of scavenging active oxygens among the flavonoids. Quercetin contains four hydroxyl groups; thus, it can act as an antioxidant and directly scavenge free radicals that are produced in the body. Quercetin treatment primarily regulates glutathione (GSH) levels to enhance antioxidant capacities. Chronic and high quercetin intake significantly increases the ratio of GSH/oxidized GSH in the liver, reduces malondialdehyde (lipid peroxidation marker) levels in the liver, adipose tissues, and small intestine, induces antioxidant enzyme expression, including glutathione peroxidase 1, catalase (CAT), and superoxide dismutase (SOD), by activating the liver nuclear factor E2-related factor 2 (Nrf2) pathway (Kobori et al., 2015). Geng et al. (2019) used the Werner syndrome (WS) human mesenchymal stem cells (hMSCs) to screen natural products and reported that quercetin could alleviate aging in hMSCs by promoting self-renewal and differentiation abilities of WS hMSCs and restoring the heterochromatin structure. *In vitro*, quercetin can promote oocyte maturation and early embryonic development in humans and aged mice by clearing mitochondrial reactive oxygen species (ROS), reducing apoptosis, and improving autophagy (Cao et al., 2020). Quercetin prolongs the lifespan and enhances motility in aged *Caenorhabditis elegans* with heat-stress tolerance by regulating insulin-like signaling and the p38-mitogen-activated protein kinase (MAPK) pathway (Sugawara and Sakamoto, 2020). Furthermore, quercetin chelates metal ions and catechol in its structure is an important antioxidant. Studies show that quercetin inhibits lipid peroxidation by binding to Fe^2+^ and attenuating iron overload and oxidative damage in chronic ethanol hepatotoxicity (Tang et al., 2014). Despite quercetin is known as a powerful antioxidant, studies have demonstrated that quercetin treatment may result in pro-oxidant, neurotoxic and cytotoxic effects depending upon certain experimental conditions (e.g., time, dose, formulation, etc.) and the pro-oxidant properties seem to be dose-dependent (Prochazkova et al., 2011). One study evaluated the effects of quercetin stabilized by microencapsulation at two doses (10 mg/kg and 100 mg/kg) on the diabetic rats. After 60 days, high-dose administration of quercetin (100 mg/kg) further aggravated the diabetic condition and resulted in harmful effects on healthy rats, pointing to a pro-oxidant activity. However, low-dose administration of quercetin (10 mg/kg) gave rise to antioxidant and protective effects on nitrergic innervation and interstitial cells of Cajal, nitrergic neurons, macrophages and oxidative/antioxidant status in diabetic rats (Vieira-Frez et al., 2020). A study investigated the potential antioxidant and prooxidant effects of quercetin on cultured non-small lung cancer A549 cells. It demonstrated concentration-dependent effects of quercetin *in vitro*, including augmentation of thiol content, total intracellular capacity at low quercetin concentrations (0.1–10 μM) and decrease in thiol content, total antioxidant capacity, cell proliferation, and induction of cell apoptosis and necrosis at higher concentrations of the quercetin (>50 mM) (Robaszkiewicz et al., 2007).

Quercetin has been regarded as an antiaging compound because of its long-term anti-inflammatory properties. The anti-inflammatory and antiallergic properties of quercetin have been proven to treat respiratory and food allergies (Escribano-Ferrer et al., 2019). It has been shown to effectively lower the levels of inflammatory cytokines in the lipopolysaccharide (LPS)-induced inflammatory RAW 264.7 cells (Kang et al., 2022) and rat intestinal epithelial (IEC-6) cells via suppressing NF-κB activation *in vitro* (Cai et al., 2021). Quercetin administration in diabetic rats also effectively decreases the levels of serum C-reactive protein (CRP), tumor necrosis factor-α (TNF-α), blood glucose, and inflammatory cytokines by enhancing AKT phosphorylation, inhibiting nuclear factor-kappa (NF-κB) levels, and reducing inflammation-induced hyperlipidemia (Hosseini et al., 2021). Elbe et al. (2016) reported that quercetin reduces the levels of inducible nitric oxide synthase (iNOS) and IL-12 in renal diseases and ameliorates the excessive accumulation of extracellular matrix and interstitial fibrosis by antagonizing NF-κB and TGF-β1/Smad2/3 signaling activation. Organ dysfunction has recently been reported to be closely related to coronavirus disease (COVID-19) progression, which may cause acute kidney injury (AKI). Quercetin has also been reported to treat COVID-19-induced AKI, which can be attributed to the anti-inflammatory properties of macrophage polarization and the inhibition of NF-κB p65 and interferon regulatory factor 5 (Diniz et al., 2020). The inhibition of proinflammatory cytokines such as IL-6, TNF-α, and IL-1β and inflammatory mediators such as catalase and nitric oxide underlie the anti-inflammatory effects of quercetin.

Several studies have found that quercetin has a spectrum of antimicrobial properties; it has significant inhibitory effects on both fungi and bacteria mainly because it can improve cell permeability, reduce enzyme activity, destroy the cell wall structure, and inhibit nucleic acid synthesis (Wang et al., 2018; Yang et al., 2020). Furthermore, in terms of the potential harm with antibiotic abuse, quercetin directly affects various microorganisms in the intestine. It can effectively enhance the diversity of gut microbiota and restore its composition after antibiotic treatment (Shi et al., 2020). Quercetin treatment also significantly improves the morphology and thickness of intestinal mucosa along with enhancing the length of the villi and glands. The levels of D-lactate and the activity of diamine oxidase were significantly decreased in the serum of quercetin-treated mice. Thus, quercetin may act as a prebiotic to combat gut dysbiosis and restore intestinal permeability. Quercetin is also a potent inhibitor of β-catenin/T-cell factor (TCF) signaling in colorectal cancer (Park et al., 2005). It has also been reported to inhibit the proliferation of colorectal cancer cells by regulating the Wnt/β-catenin signaling pathway (Shan et al., 2009). In conclusion, quercetin exhibits anti-inflammatory, antioxidant, and antimicrobial properties. It primarily regulates glutathione levels, reduces malondialdehyde levels, induces antioxidant enzyme expression, and can promote oocyte maturation. Quercetin can alleviate aging in mesenchymal stem cells and inhibit the proliferation of colorectal cancer cells. However, its effectiveness varies with experimental conditions and doses.

#### 2.1.2 Luteolin

Luteolin is a natural flavone originally isolated from *Reseda luteola* (Chen Y. et al., 2022). It is found in the leaves of many medicinal plants and foods, such as parsley, celery, broccoli, rosemary, cardamom, anise, and dandelion (Jia et al., 2015). Although luteolin is a minor component in daily nutrition, accumulating data suggest it has numerous health benefits including potent antioxidant, anti-inflammatory, and antiallergic properties.

Luteolin can prevent or reduce photoaging, which is collagen and elastin degradation, leading to wrinkled skin. It reduces the solar ultraviolet-induced release of proinflammatory cytokines (IL-6, TNF-α, and IL-20) from keratinocytes and fibroblasts, which may be attributed to its radical scavenging and antioxidant properties. Cyclooxygenase 2 (COX-2) is crucially involved in ultraviolet radiation B-induced acute inflammation by catalyzing the production of the prostaglandin precursor prostaglandin E2 (PGE_2_), and luteolin can inhibit the syntheses of COX-2 and PGE_2_. Further *in vitro* and *in vivo* studies indicate that luteolin can also modulate the SIRT3/ROS/MAPK signaling pathway to prevent skin photoaging (Mu et al., 2021), which indicates that luteolin may prevent the development of skin aging and skin cancer (Gendrisch et al., 2021). Luteolin has protective effects against vascular inflammation and inhibits monocyte binding to endothelial cells by suppressing the IκBα/NF-κB pathway (Jia et al., 2015). Luteolin pretreatment inhibited the formation of BV-2 microglia after LPS stimulation. *In vivo* results suggest that the consumption of luteolin improves spatial working memory and cognition by reducing microglia-associated inflammation in the hippocampus (Jang et al., 2010). Luteolin can also inhibit the transcription (STAT) signaling pathway to modulate the interferon-gamma-induced microglial CD40 expression (Rezai-Zadeh et al., 2008). With the progression of age, mitochondrial function, morphological content, and oxidative phosphorylation abilities appear impaired (Koopman et al., 2012). In *Caenorhabditis elegans* models, luteolin treatment improves mitochondrial dysfunction in primary neurons and enhances locomotory activities. It can also be used to regulate mitochondrial endoplasmic reticulum coupling and has potential therapeutic effects on various aging-related diseases (Naia et al., 2021). Luteolin has also been reported to increase the deacetylation of SIRT6, regulate the expression of other SIRTs and Forkhead box O3a (FOXO3a) in human monocytes, and suppress ROS production under hyperglycemic conditions (Kim et al., 2017; Akter et al., 2021).

#### 2.1.3 Epigallocatechin-3-gallate (EGCG)

Catechins are flavanols with green tea as the main component, which is a nonfermented tea extracted from the leaves of *Camellia sinensis* (Ouyang et al., 2020). Other foods or botanical drugs also contain these polyphenolic compounds, such as apples, cacaos, berries, persimmons, and grapes. Many studies have indicated that catechins have multiple health-related functions due to their antioxidative properties, anti-inflammatory properties, lipid metabolism, energy metabolism, autophagy, and immunoregulatory effects (Ouyang et al., 2020; Chen Y. et al., 2022). Catechins include EGCG, epigallocatechin, epicatechin, epicatechin-3-gallate, gallocatechin gallate, and gallocatechins. EGCG is the major and most abundant catechin (Khan and Mukhtar, 2018).

Early studies have revealed that EGCG can extend the lifespan of healthy rats by reducing damage to liver and kidney function (Niu et al., 2013). More recent studies indicate that EGCG significantly prolongs the lifespan of high-fat-diet-induced obese rats by improving free fatty acid metabolism, inflammation, and oxidative stress. It can directly increase the longevity of mRNA and protein levels of Sirt1 and FOXO1 and decrease the expression of NF-κB, acetyl-CoA carboxylase, and fatty acid synthase (Yuan et al., 2020). Consistent with these findings, green tea extracts can reverse the fat-induced mortality in *Drosophila melanogaster*, and EGCG can enhance the fitness and lifespan of *Drosophila* (Li et al., 2008; Wagner et al., 2015). EGCG supplements improve the oxidative stress in PC12 cells induced by 1-methyl-4-phenyl-pyridine *via* the SIRT1/PGC-1α signaling pathway, which provides a novel target to prevent and treat Parkinson’s disease (Ye et al., 2012). All green, puer, and black tea extracts can increase the lifespan of *Caenorhabditis elegans*, postpone beta-amyloid (Aβ)-induced progressive paralysis in transgenic worms with Alzheimer’s disease (AD), and improve the oxidative stress tolerance of worms (Fei et al., 2017). Green tea extracts have been reported to increase the number of SH-groups leading to reduced GSH accumulation in the plasma, restrained ROS generation, and improved antioxidant enzyme activities (Bártíková et al., 2015). The antioxidative effects of catechins may not be entirely manifested in SOD scavenging. Epicatechin intake can enhance Nrf2 and heme oxygenase-1 (HO1) protein levels in the nuclei of cortical neurons in mice (Wu et al., 2006), and activate the phosphatidylinositol-3-kinase/protein kinase B (PI3K)/AKT and extracellular-regulated kinase (ERK) signaling pathways in hepatocytes (Si and Liu, 2014). EGCG can also prevent stroke damage by activating Nrf2 and HO1 productions. Green tea polyphenols ameliorate early renal damage induced by a high-fat diet *via* the ketogenesis/SIRT3 pathway (Yi et al., 2017).

EGCG is regarded as a healthy and natural dietary supplement. However, EGCG also exhibits significant pro-oxidant effects under high-dose conditions (Ouyang et al., 2020). Low doses (50–300 µM) of EGCG can extend the lifespan of *Caenorhabditis elegans* and *Drosophila*, but high doses (1,000 μM) are harmful to longevity; this is because of excessive ROS accumulation and DAF-16 (a forkhead transcription factor) activation. Theanine is known to alleviate the adverse effects caused by high EGCG doses (Peng et al., 2021). The pro-oxidant properties of EGCG can be beneficial or harmful. Auto-oxidation of EGCG generates substantial ROS, which may induce apoptosis and inhibit the growth of adipocytes in cancer cells, leading to anticancer and antiobesity properties (Ouyang et al., 2020). Taken together, EGCG has antioxidant and anti-inflammatory properties and can improve lipid and energy metabolism, autophagy, and immune system regulation EGCG can also ameliorate early renal damage and prevent stroke damage. However, high-dose EGCG can exhibit significant pro-oxidant effects, leading to potential toxicity. Therefore, although green tea extracts and EGCG have many health benefits during aging, further studies are required to determine their potential toxicity, optimal doses, and applications in the field of antiaging.

#### 2.1.4 Resveratrol

Resveratrol (3, 5, 4′-trihydroxystilbene) is a polyphenol subgroup widely found in various foods and beverages consumed daily, including grapes, peanuts, pomegranates, blueberries, cocoa, and red wine. *Polygonum cuspidatum* is the plant source that is richest in resveratrol. Resveratrol was originally separated in 1940 from the roots of *Veratrum grandiflorum*, a Chinese herbal medicine, and was identified as the most important functional red wine ingredient in 1992 (Perrone et al., 2017). Studies have revealed that resveratrol exhibits a variety of pharmacological activities such as antioxidant, anti-inflammatory, antimicrobial, immunomodulatory, and neuroprotective properties; moreover, it combats age-related degenerative diseases (Diepvens et al., 2007; Perrone et al., 2017; Santhiravel et al., 2022). Considering these multiple properties, studies on the application of resveratrol as a nutritional supplement are gradually increasing.

Resveratrol is a strong antioxidant and can protect cell biomolecules from oxidative damage by directly scavenging ROS and enhancing glutathione s-transferase (GST) levels (Truong et al., 2018). Resveratrol attenuates acute kidney inflammation and oxidative stress by activating the Nrf2 pathway, restoring antioxidant capacity, reducing apoptosis, and inhibiting the NF-κB pathway and caspase cascade (Uddin et al., 2021). Dietary resveratrol has also been suggested to ameliorate H_2_O_2_-induced liver injury in tilapia along with reducing the lipid peroxidation level, inflammatory response, and immunotoxicity. Resveratrol treatment also enhances the HO-1, NAD(P)H:quinone oxidoreductase 1, GST, and IL-10 levels and decreases the inflammatory cytokine levels of IL-1β, TNF-α, and IL-8. It can modulate the Nrf2 and TLR2/MyD88/NF-κB signaling pathways (Boccellino and D'Angelo, 2020; Uddin et al., 2021). Evidence suggests that resveratrol can be considered a prebiotic to improve gut microbiota-related disorders in age-related diseases. The oral administration of resveratrol can improve glucose homeostasis in high-fat-diet-induced obese mice (Sung et al., 2017). Furthermore, obese mice receiving fecal microbiome transplants from resveratrol-fed donors have shown an improvement in metabolic syndrome within 11 days. Resveratrol fecal transplants also decrease inflammation in the colons of obese individuals (Kim et al., 2018).

Resveratrol is an activator of SIRT1, regulating cellular energy metabolism and mitochondrial homeostasis. AMPK has been reported to be an attractive target for extending a healthy lifespan, and studies show that resveratrol can activate AMPK and possesses antiobesity abilities (Si and Liu, 2014). Mice that were fed a high-fat diet containing 0.1% resveratrol showed brown-like adipocyte formation in white adipocyte tissues, along with an increased expression of uncoupling protein 1, PR domain-containing 16, cell death-inducing DFFA-like effector A, peroxisome proliferator-activated receptor-γ coactivator 1α (PGC1α), and other brown adipocyte markers. The mice also exhibited enhanced AMPK alpha1 phosphorylation (Wang et al., 2015). Studies have revealed that resveratrol and its metabolites can permeate the blood–brain barrier. Treatment of patients with mild–moderate AD with resveratrol maintains the integrity of the blood–brain barrier and contributes to cognitive and functional improvement by reducing the expression of matrix metalloproteinase-9 and inducing adaptive immune responses (Moussa et al., 2017). Resveratrol supplements in the diet increase metabolic rate, insulin sensitivity, mitochondrial biogenesis, and physical endurance in mice; they also improve the accumulation of fat and cognitive deficits of AD (Porquet et al., 2013; Moussa et al., 2017; Orlandi et al., 2017). Moreover treatment with resveratrol prolongs the mean and maximal lifespan of SAMP8 mice, a model of age-related AD, by activating the AMPK and SIRT1 pathways (Porquet et al., 2013; Bhullar and Hubbard, 2015). Resveratrol supplements are shown to extend the lifespan of *Nothobranchius guentheri* (a new model organism for aging studies), enhance the cognitive and locomotor activities, and delay the activation of senescence-associated markers when compared with the control group without affecting its body size (Yu and Li, 2012). Resveratrol treatment during later years in life has been proven to extend the lifespan and age-related symptoms of many model organisms, such as yeast, *Caenorhabditis elegans*, and *Drosophila* (Lee et al., 2016; Orlandi et al., 2017; Khan M. et al., 2019). However, recent evidence has been inconsistent. A study reveals that long-term resveratrol treatment reduces the age-related oxidative damage to DNA, lipid, and protein. Still, the study also suggests that a 12-month resveratrol intake may induce renal toxicity (Wong et al., 2009). Another study mentioned that red wine intake or oral resveratrol could delay vascular aging but does not affect the lifespan of rats (da Luz et al., 2012). A clinical randomized controlled trial also indicated that resveratrol treatment does not improve glucose and lipid metabolism or the inflammatory status of middle-aged men with metabolic syndrome (Kjær et al., 2017). Therefore, further studies are required regarding the dosage and duration of resveratrol consumption. Aging is the result of many factors in the body and these findings indicate that resveratrol is a potential antiaging therapy due to its abilities to suppress oxidative stress, inhibit inflammation, and regulate mitochondrial function and apoptosis (Zhou et al., 2021).

#### 2.1.5 Curcumin

Curcumin is a natural dietary polyphenol in *Curcuma longa* exhibiting various biological and pharmacological properties including antioxidant, immunomodulatory, anti-inflammatory, and antimicrobial properties (Abrahams et al., 2019; Khan H. et al., 2019). The chemical structure of curcumin is 1,7-bis(4-hydroxy-3-methoxyphenyl)-1,6-heptadiene-3,5-dione. It has predominantly pro-health benefits and is regularly used as a cadioprotective, nephroprotective, hepatoprotective, antineoplastic, antirheumatic, and antiaging natural plant compound (Abrahams et al., 2019; Benameur et al., 2021). Curcuminoids have been approved by the US Food and Drug Administration (FDA) as “Generally Recognized As Safe” (GRAS), and good tolerability and safety profiles have been shown by clinical trials, even at doses between 4,000 and 8,000 mg/day and of doses up to 12,000 mg/day of 95% concentration of three curcuminoids: curcumin, bisdemethoxycurcumin, and demethoxycurcumin (Lao et al., 2006). Toxicity studies have claimed that curcumin is a safe compound even at high doses (Hewlings and Kalman, 2017). Sudeep et al. (2021) confirmed the safety of a higher dose of turmeric extract/curcumin (500 mg/d) over a longer duration (90 days) in healthy subjects and opposed the possibility of any hepatic damage caused by turmeric/curcumin supplementation. However, current research suggests some potential risks with its use. Luber et al. (2019) reported two case studies indicating liver toxicity due to the consumption of turmeric supplements. A study performed a comprehensive assessment of the effects of parenteral administration of turmeric extract (intraperitoneal) with low (100 mg/kg) and high doses (250 mg/kg) in comparison to oral curcumin (500 mg/d) administration in a streptozocin (STZ)-induced diabetic rat model, and found that low and high doses of turmeric showed protective effects histologically on the pancreas and the kidneys, high doses both injected and orally administered caused an elevation of liver enzymes and liver damage (Essa et al., 2019). According to the World Health Organization and Food and Agriculture Organization, the recommended maximum intake of curcumin is 1 mg/kg body weight (Soleimani et al., 2018). Several studies have reported that curcumin can prevent and treat several age-related diseases. Studies have also shown that curcumin can remarkably extend lifespan and promote healthy aging in mice, *Caenorhabditis elegans*, yeasts (Stępień et al., 2020), and *Drosophila* (Suckow and Suckow, 2006; Esquivel et al., 2020). The mechanisms underlying curcumin-related effects on biological aging have been proposed, including inducing autophagy, inhibiting inflammation and oxidation, stimulating AMPK and SIRT1 pathways, inhibiting the mammalian target of rapamycin (mTOR) signaling pathway, and maintaining mitochondrial function (Benameur et al., 2021).

Curcumin also strongly prevents oxidative stress and it can protect against lipid and protein oxidation. Furthermore, it can eliminate ROS, reduce the levels of malondialdehyde (MDA), protein carbonyls, thiols, and nitrotyrosines, and stimulate the activities of SOD and catalase. A meta-analysis of randomized controlled trials suggested that exposure to curcumin for ≥4 weeks significantly reduced the MDA levels in serum, glutathione peroxidase activity in erythrocytes, and increased SOD activity (Qin et al., 2018). Rahman et al. investigated the effects of curcumin on oxidative stress in a D-galactose and naturally-induced aging mouse model. Curcumin improved retention and freezing memory and ameliorated the levels of oxidative stress biomarkers (ROS and RNS) in the D-galactose and naturally-induced aging mice. The antiaging effects of curcumin may be attributed to the upregulation of antioxidant enzymes through binding with glutathione S-transferase A1, glutathione S-transferase omega-1, kelch-like ECH-associated protein 1, and inhibition of oxidative damage through binding with beta-secretase 1 and amine oxidase [flavin-containing] A (Rahman et al., 2022). Another study reported that curcumin attenuated H_2_O_2_-induced oxidative stress in RAW264.7 cells by increasing the activities of antioxidant enzymes (CAT, SOD, and GSH-PX) and activating the Nrf2-Keap1 signaling pathway (Meng et al., 2021). Curcumin also antagonizes oxidative stress in blood vessels, muscles, and liver by upregulating SIRT1 and Nrf2 and downregulating the p53/p21 pathway (Ren et al., 2019; Cox et al., 2022). Clinical and nutritional epidemiological studies have revealed that the long-term ingestion of curcumin can prevent cardiovascular disease and reduce pathological factors triggering cardiovascular diseases (Hong et al., 2019). The oral administration of curcumin in spontaneously hypertensive rats significantly reduced the expression of connective tissue growth factor, type III collagen, and fibronectin in the left ventricle of the heart and upregulated peroxisome proliferator-activated receptor gamma (PPARγ) activity, thereby alleviating hypertension-induced myocardial fibrosis (Meng et al., 2014). The antioxidant effects of curcumin can reduce oxygen radicals produced *in vivo*, induce antiapoptotic effects, and resist myocardial infarction-induced cardiac dysfunction (Lv et al., 2016). Studies reporting vascular endothelial dysfunction in porcine coronary arteries have revealed that curcumin can impede homocysteine-induced endothelium-dependent vasodilatory dysfunction, reduce endothelial-type nitric oxide synthase levels, and decrease the production of superoxide anion radicals, thereby alleviating vascular endothelial dysfunction (Ramaswami et al., 2004). Altogether, these findings suggest that as a major component of the daily diet, curcumin can prevent cardiovascular disease development. The cardioprotective effects of curcumin may lie in its ability to directly influence various enzymes (Yuan et al., 2005; Potter, 2013).

Many recent studies *in vitro* and *in vivo* have proved that curcumin has protective effects against neuroinflammation (Bielak-Zmijewska et al., 2019; Lin et al., 2019; Cianciulli et al., 2022). The anti-inflammatory effects of curcumin have contributed to multiple molecular targets including NF-κB. A study revealed that curcumin treatment after LPS stimulation significantly reduced the release of inflammatory mediators and suppressed microglial TLR4/MyD88/NF-κB signaling pathway protein expressions along with neuronal apoptosis in experimental traumatic brain injury (Zhu et al., 2014). Along with suppressing the NF-κB signaling pathway in inflammatory processes, curcumin supplementation can increase the production of anti-inflammatory cytokines in murine BV-2 microglia cells *via* the JAK/STAT/SOCS signaling pathway (Porro et al., 2019). Other anti-inflammatory effects of curcumin include downregulating the levels of monocyte chemoattractant protein-1, COX-2, lipoxygenase, iNOS, MAPK, and other inflammatory mediators (Cianciulli et al., 2022). As an alternative epigenetic modulator, curcumin enhances SIRT1 expression and ultimately has protective effects against many neurological disorders (Zendedel et al., 2018). Although curcumin has several pharmacological properties against many disorders, it has poor bioavailability and low gastrointestinal absorption, which are mainly attributed to poor solubility and rapid clearance (Shabbir et al., 2021). The use of curcumin-loaded nanoparticles has been elucidated to be a safe and novel therapeutic strategy (Yavarpour-Bali et al., 2019; Tagde et al., 2021). These findings indicate that curcumin intake in elderly individuals may promote healthy aging and minimize the risk of age-related diseases. However, higher doses of turmeric and curcumin as an oral supplement should be used with caution in future studies and in clinical trials since they are potentially hepatotoxic.

#### 2.1.6 Lignans

Lignans are dimers derived from cinnamic acid and its derivatives (Rodríguez-García et al., 2019). Lignans are molecules with two phenyl propane units coupled at the central carbon of the side chain, whereas compounds with alternative coupling are called neolignans (Lu et al., 2016). Dietary sources of lignans are particularly abundant, including oilseeds (flaxseed, sesame, linseed, and sunflower), whereas other foods contain lower levels (Sun J. et al., 2018). However, dietary surveys have revealed that whole grains (especially rye), legumes, fruits, vegetables, nuts, tea, and coffee can also be considered good sources of lignans. Lignans can be classified into eight subgroups, arylnaphthalenes, aryltetralins, dibenzocyclooctadienes, dibenzylbutanes, dibenzylbutyrolactones, dibenzylbutyrolactols, furans, and furofurans, according to the cyclization and oxygen incorporation patterns (Osmakov et al., 2022). Lignans are metabolized to enterodiol and enterolactone by gut microorganisms and exhibit various biological activities, including anti-inflammatory and antioxidative properties. Lignans can also combat the progression of age-related diseases such as cancer, cardiovascular diseases, neurodegenerative diseases, and metabolic diseases (Šmejkal et al., 2010; Sun J. et al., 2018; Egawa et al., 2020; Maia et al., 2020).

Shimoyoshi *et al.* reported that sesame lignans could prevent age-related cognitive decline by reducing reactive carbonyl species production in aging-accelerated mice via antioxidant properties; thus, long-term consumption of sesame lignans might effectively prevent age-related brain dysfunction (Shimoyoshi et al., 2019). Similarly, dibenzocyclooctadiene lignans isolated from a Chinese botanical drugs, *Schisandra chinensis*, have anti-inflammatory and antioxidant properties, as well as improve cognitive function (Hu et al., 2014). High levels of dietary lignan intake significantly reduces the risk of mortality from postmenopausal breast cancer (Franco et al., 2005) and is also associated with a lower risk of other cancer types and developing cardiovascular diseases in women experiencing postmenopausal symptoms and elderly men (Pellegrini et al., 2010; Peterson et al., 2010; Lin et al., 2014). Lignans exert anti-inflammatory and antioxidative effects by inhibiting NF-κB, c-Jun N-terminal kinase (JNK)/p38 MAPK, and PI3K/Akt signaling pathways and activating the antioxidant Nrf2/HO-1 pathway (Osmakov et al., 2022). Nectandrin B, a bioactive lignan isolated from nutmeg, reduces cellular oxidative stress by directly scavenging free radicals or indirectly inducing antioxidant enzyme expression by activating AMPK, thereby reducing cellular senescence. It significantly reduces H_2_O_2_- and palmitate-induced intracellular ROS production in young and elderly human diploid fibroblasts (HDFs), stimulates the expression of SOD I and II in aged HDFs, activates the PI3K/Akt pathway, reversing ERK1/2 and p38 activity (Jang et al., 2019). Lignans from guaiac resin are shown to decrease nitric oxide production in interleukin 1β-treated rat hepatocytes, exhibiting anti-inflammatory effects. Furthermore, lignans ameliorate silica-related oxidative stress, immune-related inflammatory response, and fibrosis by inhibiting TLR-4/NLRP3/TGF-β signaling and increasing mitochondrial membrane potential *in vitro* and *in vivo*. Thus, they inhibit ROS production, macrophage polarization, and myofibroblast differentiation (Liu et al., 2021). Lignans also sensitize cancer cells to treatment-induced cytotoxicity by inhibiting cell survival pathways including PI3K/Akt, NF-κB, and X-linked apoptosis inhibitory protein, and by stimulating apoptotic pathways such as tumor suppressor p53 (Saarinen et al., 2010). Current observations highlighted the bioactive properties of lignans as human health-promoting molecules. Due to their various bioactive properties, dietary intake of lignan-rich foods may prevent certain types of cancers and lower the risk of developing cardiovascular disease. Nonetheless, further human studies are warranted to evaluate lignan bioavailability resulting from different traditional dietary patterns, thus affect the rational promotion of healthy lignan-rich diets.

Bioactive polyphenols, such as quercetin, luteolin, EGCG, resveratrol, curcumin, and lignans have a substantial impact on the aging process in different organisms. Polyphenols have some advantages compared to chemical inducers of autophagy due to their intrinsic natural bio-compatibility and safety. In this context, polyphenols can be used as part of the diet or as separate compounds (supplements) that have potential therapeutic effects on healthy aging (Ullah and Khan, 2018; Yessenkyzy et al., 2020). However, the clinical efficacy and potential toxicity of high dose intake of polyphenols need to be further studied and validated in the near future.

### 2.2 Saponins

Saponins are glycosides with triterpenoids or spirostanols, which are widely found in terrestrial higher plants, including *Panax ginseng*, *Gynostemma pentaphyllum,* and *Glycyrrhiz*a, and in few marine organisms such as starfish and sea cucumbers. Saponins are roughly divided into two categories based on their aglycone structures: triterpenoid saponins (1) and steroidal saponins (2) (Haralampidis et al., 2001). Saponins are the main functional components of many plant drugs and folk medicines; they are also the primary components contributing to many pharmacological properties (He et al., 2019). Saponins have primary functions of enhancing bidirectional immune regulation (Rajput et al., 2007), reducing cholesterol (Matsuura, 2001), and exhibiting antitumor activities (Elekofehinti et al., 2021). The bioavailability of saponins after oral administration is relatively low; however, biotransformation can be used to obtain rare saponins with relatively high bioavailability. Biotransformation involves the structural modification of saponins to efficiently obtain the steady target compounds. There are several natural saponins, including ginsenosides, gypenosides, glycyrrhizins, saikosaponins, dioscins, timosaponins, astragalosides, and ardipusillosides (He et al., 2019).

Ginsenosides are a class of active components extracted from ginseng (such as *Panax ginseng*, *Panax quinquefolium*, and *Panax notoginseng*). Rb1, Rb2, Rc, and Rd have more glycosyl groups on C-3 and C-20 than rare ginsenosides, including F2, Rg3, Rh2, and C-K (Hou et al., 2021; Abduljawad et al., 2022). Therefore, deglycosylation and hydrolysis can be used to prepare rare ginsenosides with high activity to reduce the number of glycosyl groups in ginsenosides. Various ginsenosides play critical roles in inhibiting oxidative stress, preventing oxidative injury, and protecting cells. Recent studies have reported the biological effects of ginsenosides in cell culture or animal models. Animal studies have revealed that ginsenosides have beneficial effects when treating the pathological conditions of different tissues. Ginsenosides can ameliorate diverse pathogenic conditions, which can be attributed to their effects on the production of ROS. These substances primarily affect the activity of the AMPK/Akt and PI3K/Akt pathways (Ghafouri-Fard et al., 2022). Of all available ginsenosides isolated from the genus *Panax*, antiaging intervention studies have focused on ginsenosides Rb1, Rd, Re, Rg3, and Rg1 (Zhang et al., 2021). The antiaging effects ginsenosides have been reported primarily in rodents and worms, and their broad antiaging mechanisms are usually attributed to their antioxidant, anti-inflammatory, antiapoptotic, and mitochondrial protective effects, along with activating trophic-sensing response signals, inducing autophagy, and preventing cellular senescence. For example, ginsenoside Rg3 exerts neuroprotective effects in rotenone-induced Parkinson’s disease mice *via* antioxidative properties (Han et al., 2021). Ginsenoside Rg1 significantly protected H9c2 cardiomyocytes from post-hypoxia/reoxygenation injury (Zhu et al., 2009). Gypenosides are the most important functional components of *Gynostemma pentaphyllum*, exhibiting antiaging, anticancer, hypoglycemic, lipid-lowering, and neuroprotective properties (Subramaniyam et al., 2011). Alfalfa saponins can rescue cell viability, elevate the activity of antioxidant enzymes, and downregulate the activities of LDH and MDA in H_2_O_2_-induced cells. They can also inhibit oxidative stress-induced cell mitochondrial apoptosis *via* the MAPK signaling pathway (Cui et al., 2022). *Panax notoginseng* total saponin (PNS) content can significantly enhance the levels of SOD, CAT and GSH, and high doses of PNS can reduce cholesterol levels, indicating that high doses of PNS can lower blood lipids and prevent atherosclerosis, reducing age-related cardiovascular diseases and thus prolonging life (Kim and Lee, 2010). Furthermore, PNS content can modulate gut microbiota to promote thermogenesis and beige adipocyte reconstruction *via* leptin-mediated AMPKα/STAT3 signaling in mice with diet-induced obesity (Xu et al., 2020). Sea cucumber saponins (SCSs) have also been reported to be highly effective in alleviating obesity-induced inflammation and insulin resistance better than regular saponins. They significantly ameliorate glucose and lipid disorders by inhibiting fatty acid biosynthesis. SCSs also promote hepatic glycogen synthesis and reduce insulin resistance by upregulating p-GSK3 levels in liver cells (Han et al., 2019). SCS liposomes can reduce the release of pro-inflammatory cytokines and macrophage infiltration; they can also effectively influence the p-ERK/cPLA2/COX1 pathway and ultimately reduce PGE2 levels in adipose tissues (Chen et al., 2017).

Natural sources of saponins have long been used in herbal and traditional medicine. However, due to the scarcity of quantity and structural heterogeneity, separating saponins from nature is difficult and laborious. Unlike other natural products, the bioavailability of original saponins after oral administration is relatively low, which restricts their functions. Chemical synthesis and biotransformation pathways of natural saponins are gradually discovered, which is helpful to enrich the structural diversity of saponins and develop promising compounds (Juang and Liang, 2020).

### 2.3 Alkaloids

Alkaloids are one of the most important classes of natural products exhibiting structural diversity and significant pharmacological effects (Bhambhani et al., 2021). They are widespread in higher plants such as Berberidaceae, Amaryllidaceae, Liliaceae, Leguminaceae, Papaveraceae, Ranunculaceae, and Solanaceae families (Kaur and Arora, 2015). A large number of monoterpenoid indole and bisindole alkaloids have been identified in the genus *Alstonia* to date. There are three main types of alkaloids: true alkaloids, protoalkaloids, and pseudoalkaloids. True alkaloids are obtained from amino acids and they have a nitrogen-containing heterocyclic ring. Cocaine, morphine, and quinine are true alkaloids commonly found in nature. Protoalkaloids contain a nitrogen atom and are derived from amino acids but are not part of the heterocyclic system and mainly include yohimbine, mescaline, and hordenine. L-tryptophan and L-tyrosine are the primary precursors of this alkaloid group. The basic carbon backbone of pseudoalkaloids is not derived from amino acids directly, and commonly found types are capsaicin, caffeine, and ephedrine. The clinical applications of many alkaloids are similar to their traditional and folkloric applications. For example, berberine (BBR) is the primary component of BBR in the rhizomes of *Coptis chinensis*, and it has antibacterial and anti-inflammatory properties. The morphine base contained in poppy rind is an effective analgesic. Quinine is a valuable antipyretic. Trichothecene and vincristine have highly effective anticancer properties.

Caffeine is a purine alkaloid and is among the most popular and widely consumed beverages worldwide. It is mainly derived from coffee; however, it may also be found in other plants including tea, guarana berries, and cocoa beans. Caffeine is also found in certain energy drinks, soft drinks, chewing gum, and medicines (Gonzalez de Mejia and Ramirez-Mares, 2014). Caffeine is commonly used to stay awake and alert, improve cognitive performance and concentration, and reduce fatigue (Walter, 2022). In addition, research is being done on whether caffeine can reduce cognitive impairment in age-related disorders. Epidemiological studies have reported an association between chronic caffeine intake and a significantly low risk of developing neurodegenerative diseases, such as AD (Eskelinen et al., 2009). Similarly, in experimental models of AD, chronic caffeine treatment has been effective in preventing beta-amyloid (Aβ) production and memory deficits (Espinosa et al., 2013). A recent study indicated that caffeine could promote telomerase reverse transcriptase expression at mRNA and protein levels, and it consequently extended telomere length and prevented cellular senescence (Tao et al., 2021). *In vitro* results revealed that caffeine can safely and effectively control or delay oocyte aging and maintain the quality of aged oocytes in mice (Zhang et al., 2017). In an aged *Caenorhabditis elegans* model, the long-term intake of caffeine (10 mM for 3 days at 25°C) in L4 stage worms inhibited actin-5 mislocalization, reduced autophagy levels in senescent animals, improved mitochondrial function, and increased oxidative stress resistance and antioxidant protein expression (Min et al., 2021). Altogether, these findings revealed that caffeine is a potential antiaging agent. However, the cognition-enhancing properties of caffeine in aged individuals remain controversial (Shukitt-Hale et al., 2013; Cappelletti et al., 2015). For example, study reported that long-term treatment with low doses of caffeine exacerbated neuropsychiatric symptoms, called behavioral and psychological symptoms of dementia in 3xTg-AD mice models of AD (Baeta-Corral et al., 2018). Some reports also indicated that caffeine and dobutamine can induce ventricular tachyarrhythmias in normal rats. Caffeine also induced spontaneous ventricular arrhythmias in normal rats, especially in older rats, and no sex-related differences were noted in caffeine-induced arrhythmia (Chen V. et al., 2022). Nwabuo et al. (2020) also showed that long-term coffee intake could exacerbate generalized anxiety disorders and left-ventricular function. Some 95% of the US adult population consume caffeine, and the general perception is that there are no negative consequences for health. The upper limit of safe consumption is less than 400 mg per day (Hamed, 2018). Previous studies suggested that the ingestion of 3–9 mg/kg (200–600 mg/d) of caffeine seems to be generally safe (Grgic et al., 2019). Toxic effects occur when intake reaches or exceeds 1.2 g, and a dose of 10–14 g can cause death (van Dam et al., 2020). Although the evidence for the adverse effects of caffeine on fetal health is inconclusive, caution is recommended to limit caffeine intake to 200 mg/day during pregnancy (Tetens, 2015).

Various bitter ginseng alkaloids have remarkable functions in cardiac strengthening and antiarrhythmic aspects. Matrine is an alkaloid from the genus *Sophora flavescens* and has antidiabetic properties, antitumor effects, ameliorates isoproterenol-induced heart disease, and has neuroprotective effects. Sun et al. recently reported that MAT attenuated D-galactose-induced age-related cognitive impairment and memory deficits by inhibiting cellular senescence and oxidative stress (Sun K. et al., 2018).

Chelerythrine is a benzophenanthridine alkaloid that is extracted from *Chelidonium majus* L., *Macleaya cordata* (Willd.) R. Br., *Sanguinaria canadensis* L., and *Zanthoxylum asiaticum* (L.) Appelhans, Groppo & J. Wen (Wang et al., 2013). It has various biological properties, such as anticancer, antidiabetic (Zheng et al., 2015), antifungal (He et al., 2018), and antiparasitic properties (Li et al., 2011). Chelerythrine exerts excellent antitumor activity by inducing cancer cell apoptosis and inhibiting telomerase activity, tumor invasion, and metastasis (Chen N. et al., 2022).

Galanthamine is a phencyclidine alkaloid and a reversible acetylcholinesterase inhibitor that inhibits acetylcholinesterase activity in a long-term and competitive manner. Jiang et al. (2018) reported that galanthamine protects neurons by inhibiting NOX4 expression and accumulating ROS, ultimately inhibiting Aβ-induced autophagy in PC12 cells. Another study also suggested that it can be utilized in the treatment of cognitive dysfunction occurring in AD or during the normal course of aging (Lamirault et al., 2003).

Berberine (BBR) is an important quaternary benzylisoquinoline alkaloid with a wide spectrum of pharmacological actions, including hypoglycemic, hypolipidemic, anticancer, antiobesity, hepatoprotective, anti-inflammatory, and antioxidant activities (Neag et al., 2018; An et al., 2022). It is primarily extracted from the roots, rhizomes, and stem bulks of the Berberidaceae and Ranunculaceae families (including *Hydrastis canadensis*, the Chinese botanical drug Huanglian, and several other plants) (Feng et al., 2019). BBR has been shown to protect against renal aging. BBR can protect against D-galactose-induced renal aging in rats by downregulating PTEN expression and enhancing Akt activity (El-Horany et al., 2020). BBR has been reported to markedly improve aging-related manifestations such as cognitive impairment and muscle dysfunction, and the study indicated that AMPK/SIRT1/PGC1α pathway activation in skeletal muscle might be the underlying protective mechanism for the antiaging effects of BBR (Yu et al., 2018). BBR treatment can reduce ROS levels in endothelial cells after acute exposure to H_2_O_2_ *in vitro*. Furthermore, BBR reduced REDD1 expression to interrupt the ROS-DNA damage response positive feedback loop and restore autophagic flux, ameliorating the senescence of retinal pigment epithelium (Chen Q. et al., 2022). BBR also inhibits NLRP3 inflammasome activity and IL-1β production induced by saturated fatty acids (palmitate) in adipose tissue-derived macrophages by activating AMPK-dependent autophagy (Zhou et al., 2017). Therefore, BBR is a potential antiaging natural plant compound; however, its low oral bioavailability limits its clinical applications. The use of polymer materials and nanotechnology provides novel ways to improve its bioavailability (Xu et al., 2019).

Alkaloids are naturally occurring specialized metabolites with nitrogen as a characteristic element present in their chemical structures. Among natural products, phenolic compounds and alkaloids are multi-target drugs with the strongest antioxidant, anti-inflammatory effects, neurotrophic effects and have the potential of high efficiency and low toxicity against aging and age-related diseases (Kooshki et al., 2023). However, more studies are needed to prove the efficacy and possible side effects of alkaloids.

### 2.4 Polysaccharides

Polysaccharides are active substances widely found in plants, animals, and microorganisms. Various natural polysaccharides extracted from functional and medicinal foods have attracted attention over the last decade for their biological safety and beneficial roles in preventing chronic diseases such as cancer, diabetes, and neurodegenerative disorders. Natural polysaccharides can also delay aging by reducing oxidative damage and inflammatory effects and inhibiting telomere shortening in different aging models (Table 2).

**TABLE 2:** Representative polysaccharides and their bioactive substances related to antiaging activity.

Natural product	Model	Antiaging mechanisms	Outcomes	References
*Polygonatum sibiricum* polysaccharides	Rats	Activating D-gal	↑ SOD, GSH-Px	Zheng (2020)
			↓ MDA	
			↓ β-galactosidase	
			↑ FOXO3, AKT	
*Cordyceps cicadae* polysaccharides	*Drosophila*	Antioxidant properties	↑ CAT, SOD1 and MTH	Zhu et al. (2020)
Neutral polysaccharides from *Rehmannia glutinosa*	*Caenorhabditis elegans*	Insulin/IGF-1 signaling pathway	↑ SOD, CAT	Yuan et al. (2019b)
			↓ lipofuscin expression	
Polysaccharides extracted from ginsenoside residues	*Caenorhabditis elegans*	Antioxidant properties	↓ lipofuscin expression	Sun et al. (2023)
			↓ ROS	
			↑ SOD	
*Athyrium multidentatum* (Doll.) Ching polysaccharides	Aging mice	Activating the PI3K/Akt/Nrf2 and FOXO3a pathways	↑ PI3K, AKT, Nrf2,	Jing et al. (2019)
			↑ FOXO3a, OH-1	
			↓ caspase-3	
Polysaccharides of *Cornus officinalis*	Aging mice	Reducing the rate of cellular senescence	↓ caspsae-3, Bax	Wang et al. (2019)
*Lycium barbarum* polysaccharides	*Caenorhabditis elegans*;	Activating the IIS pathway	↑ Sir-2.1, daf-16	Chen et al. (2009), Xia et al. (2014), Zhang et al. (2019)
	Zebrafish		↓ p21, p53	
*Angelica sinensis* polysaccharides	Nestin-GTP transgenic mice	Regulating the p53 signaling pathway	↓ p21, p53	Cheng et al. (2018)
*Dendrobium officinal* polysaccharides	Mice	Inhibiting inflammation and oxidation	↑ TAOC, SOD, GSH-Px	Wu et al. (2018)
			↑ IL-10, Bcl-2/p53	
			↓ IL-6, IL-12	
			↓ MDA	

*Polygonatum sibiricum* polysaccharides significantly increase SOD and GSH-Px activities, inhibit MDA levels and β-galactosidase activity in the kidney tissues of D-galactosamine-induced aged rats, and increase FOXO3 and Akt protein expressions in kidney tissues, thus exerting antiaging effects (Zheng, 2020). *Cordyceps cicadae* polysaccharides may prolong the lifespan of *Drosophila* by upregulating the expressions of antioxidant genes CAT, SOD1, and MTH (Zhu et al., 2020). Neutral polysaccharides from *Rehmannia glutinosa* (NPRG) extended the lifespan and delayed senescence of *Caenorhabditis elegans* along with increasing SOD and CAT activities, scavenging excess ROS, and reducing lipofuscin expression in the worms. Study results also revealed that the life-extending effects of NPRG were associated with the IIS pathway and could promote the nuclear localization of DAF-16 (Yuan Y. et al., 2019). Polysaccharides extracted from treatment with ginsenoside residues could prolong the mean lifespan of *Caenorhabditis elegans* by 58.60% without affecting its locomotive behavior. Simultaneously, ginsenoside residues decreased the levels of lipofuscin and ROS, and increased SOD activity, thus preventing the aging-related oxidative damage (Sun et al., 2023).

The protein activities of Bax and caspase-3 (the most critical apoptosis-executing protease during apoptosis, which cleaves the Bcl-2 protein and triggers apoptosis upon activation) are elevated and Bcl-2 activity is reduced in senescent mouse cells (Rempuia et al., 2022). *Athyrium multidentatum* (Doll.) Ching polysaccharides showed effective antiaging properties by activating the PI3K/Akt/Nrf2 and FOXO3a pathways, increasing phosphatidylinositol 3-kinase (PI3K), Akt, nuclear factor-erythroid 2-related factor 2 (Nrf2), FOXO3a, and HO-1 expression, increasing the Bcl-2/Bax ratio, and inhibiting caspase-3 expression (Jing et al., 2019). Wang *et al.* reported that the oral administration of polysaccharides of *Cornus officinalis* significantly restored Bcl-2 protein levels and decreased the expressions of Bax and caspase-3 in the ovarian granulosa cells of senescent mice, indicating that these polysaccharides could prevent apoptosis in senescent mice ovarian granulosa cells and reduce cellular senescence rates (Wang et al., 2019). Plant polysaccharides are also involved in enhancing the immunity of the body. For instance, *Lycium barbarum* polysaccharides (LBPs) can enhance the responses of T helper cells 1 and T helper cells 2 in dendritic cells (Chen et al., 2009). Zhang et al. (2019) reported that the lifespan of sir-2.1 and daf-16 mutant nematodes with LBP intervention was shorter than that of normal nematodes, suggesting that LBP prolongs nematode lifespan by activating the IIS pathway and regulating the activities of sir-2.1 and daf-16 genes. Furthermore, LBP could reduce the expression of senescence-related genes p21 and p53 in zebrafish embryos (Xia et al., 2014). This indicates that LBP has important antiaging properties. Other polysaccharides that regulate the p53 signaling pathway include *Angelica sinensis* polysaccharides and *Dendrobium officinale* polysaccharides (Wu et al., 2018).

These studies suggest that polysaccharides play an important role in anti-aging due to its strong antioxidants capacity. Polysaccharides comes from a wide range of sources with few side effects on human health. More and more polysaccharides are being used to develop anti-aging functional foods. In addition, recent studies have shown that many polysaccharides can target key anti-aging molecules such as SIRT1, mTOR, AMPK and p53, and their anti-aging mechanisms seem to be solved at the genetic and molecular levels. However, there remains a lack of long-term and large-scale clinical trials of polysaccharides as potential anti-aging drugs. In the future research, it will be of great value to analyze the composition and function of polysaccharides and study its anti-aging mechanisms through clinical trials (Guo et al., 2022).

## 3 Molecular mechanisms underlying dietary supplements and natural products activities

Human aging is a complex, multidimensional and inevitable process that is characterized by a gradual and progressive loss of physiological integrity and functions (Jan et al., 2019). It is a recognized major risk factor that increases susceptibility to human diseases, especially in the elderly adults. As we age, functional and biological decline can remarkably affect the processes of the body system, switching the balance to massive cellular alterations and organ damages leading to aging-associated disease (Catana et al., 2018). For example, the ability of the immune system to perform its intended duties has steadily declined leading to pathological changes and functional damage. Telomere length is maximum at birth and decreases progressively with advancing age and this age associated decrease in the length of telomere is linked to various ageing associated diseases (Rizvi et al., 2014). Metabolic diseases are caused by disorders in substance anabolism and catabolism, which are closely correlated with aging (Ni and Liu, 2021). Neurodegenerative diseases are featured by a progressive loss of selective populations of vulnerable neurons and they and they can be classified as AD, Parkinson’s disease, or motor neuron disease according to the clinical characteristics, including cognitive impairment, neuroinflammation, neuron loss, β-amyloid accumulation, the collapse of proteostasis networks, and others (Hou et al., 2019).

**FIGURE 2:**
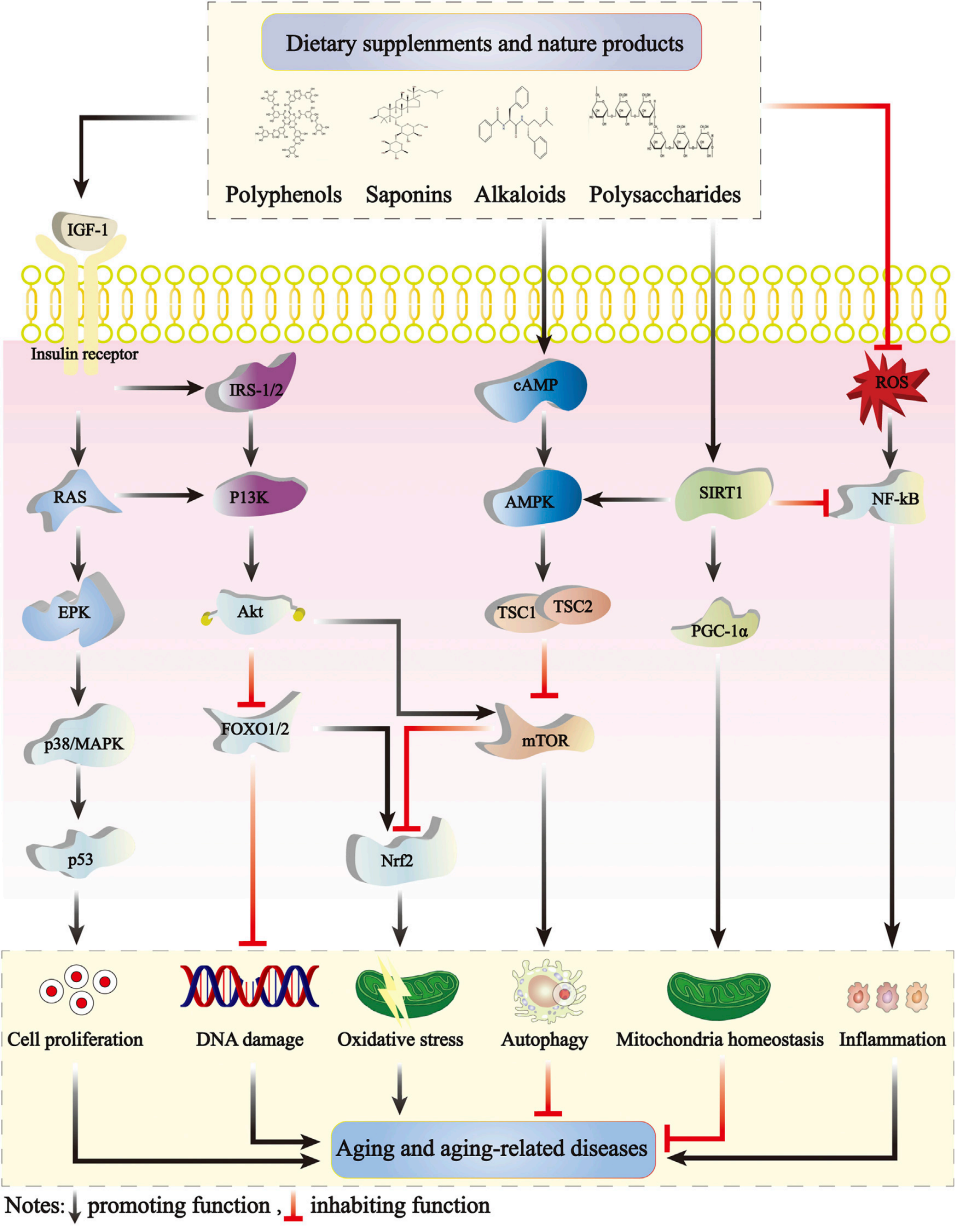
The antiaging mechanisms of dietary supplements and natural products. The IIS, mTOR, AMPK, and SIRT1 signaling pathways modulate aging by interacting with one another. The IIS signaling pathway crucially regulates aging and is the earliest defined aging-related pathway. IGF-1 initiates complex intracellular signaling cascades by binding to high-affinity IGF-1 receptors on the cell surface, activating insulin receptor substrate (IRS) molecular phosphorylation, activating the PI3K-Akt pathway and p38/MAPK signaling cascades, and controlling multiple functions including mTOR activity and FOXO translocation. The IIS signaling pathways also inhibit AMPK activation. SIRT1 negatively regulates NF-κB signaling and plays an important anti-inflammatory role. Dietary supplements and natural products can delay the development of aging by mitigating cell proliferation, DNA damage, oxidative stress, inflammation, and activating cell autophagy and mitochondrial homeostasis. AMPK, adenosine 5′-monophosphate-activated protein kinase; mTOR, mammalian target of rapamycin; NF-κB, nuclear factor kappa-B; Nrf2, nuclear factor E2-related factor 2; IIS, insulin/insulin-like growth factor 1 signaling.

The primary cellular pathways mediating longevity-extending effects of natural products involve nutrient-sensing pathways. Among these, SIRT/NAD+, AMPK, mTOR, p53, and IIS pathways are the most widely studied longevity mediators (Figure 2). The mitochondrion is a metabolic organelle, and it connects with several metabolic signaling mediators. Breakdown in mitochondrial function and metabolic signaling of the aforementioned longevity mediators typically happens over time. This breakdown disrupts metabolic health, leading to cardiovascular and metabolic disorders in elderly individuals (Natarajan, 2023). Cancer is a multifactorial disease with non-specific age-occurrence dynamics (Torre et al., 2016). However, both aging and cancer are characterized by a series of partially overlapping “hallmarks.” The equivalence or antagonism between aging-associated deregulated nutrient-sensing and cancer-relevant alterations of cellular metabolism is complex. For example, the IIS pathway mediates longevity, however, cancerous cells expropriate this pathway to aid in uncontrolled proliferation. Other features of aging, such as telomere attrition and stem cell exhaustion, act likely to suppress oncogenesis (López-Otín et al., 2023).

The IIS signaling pathway exerts a crucial regulatory function in aging and is the first established lifespan-regulating signaling pathway (Kenyon, 2011). IGF-1 triggers intricate intracellular signaling cascades by binding to high-affinity IGF-1 receptors on the cell surface and inducing phosphorylation of insulin receptor substrate molecules, as well as activating the PI3K-Akt and p38/MAPK signaling cascades. Activation of the IIS pathway inhibits several transcription factors, including DAF-16/Forkhead box O (FOXO), heat shock factor-1, and SKN-1/NRF2, thereby suppressing downstream target genes that promote longevity (Lee and Lee, 2022). Yuan *et al.* reported that two fractions of *Rehmannia glutinosa* polysaccharides (NPRR) could increase SOD and CAT activities, scavenge excess ROS, and reduce lipofuscin expression in *Caenorhabditis elegans*. The neutral polysaccharides of astragalus could also promote the nuclear localization of daf-16 and prolong *Caenorhabditis elegans* lifespan *via* the IIS pathway (Yuan Y. et al., 2019).

FOXO and p53 proteins serve as transcription factors that regulate diverse signaling pathways involved in the cell cycle, apoptosis, and metabolism control. Inflammatory factors can activate cellular aging pathways, upregulating p53 expression and subsequent activating the downstream target coding protein p21, a cyclin-dependent protein kinase inhibitor. Activation of p21 protein can impede cellular progression from the G1 phase to the S phase, causing cellular senescence (Bourgeois and Madl, 2018). Yu *et al.* found that after a 10-month intervention with ginsenoside Rb1, significant improvements were observed in the age-related physiological changes in mice, along with a reversal in the expression of proteins p53 and p21 in the heart tissue of aging mice. Additionally, the intervention affected the cell cycle progression and apoptosis, and alleviated metabolic disorders (Yu et al., 2020).

AMPK is a heterotrimeric complex consisting catalytic (α1, α2, β1, and β2) and regulatory subunits (γ1, γ2, and γ3). Different isomers of α, β, and γ are expressed in carious metabolism-related organs and can form various combinations. AMPK plays important roles in cellular energy metabolism. With an increase in the intracellular AMP/ATP ratio, AMPK phosphorylates and stimulates several downstream target molecules, thus reducing ATP utilization and enhancing ATP production. These cellular processes eventually lead to catabolism. A decrease in the AMP/ATP ratio leads to AMPK inhibition which, in turn, promotes cellular anabolism (Herzig and Shaw, 2017). The decline of AMPK activity has been reported to be associated with aging, and the reduction of AMPK activity may contribute to the onset of various age-related diseases. Simultaneously, AMPK fundamentally regulates cell growth and proliferation, establishing and stabilizing cell polarity, regulating animal lifespan, and physiological rhythm. Studies have mentioned that AMPK activity declines with age and energy imbalance is a major contributing factor to age-related diseases (Yang et al., 2019; Yang et al., 2022; Xu et al., 2023). Xu et al. (2017) reported that Rhizoma coptis and BBR are potential antiaging agents, which promote healthy aging *via* antioxidative properties and AMPK activation, prolonging the lifespan of various organisms and alleviating age-related diseases.

mTOR is an atypical serine/threonine protein kinase belonging to the PI3K-associated kinase family that interacts with several proteins to form two different complexes: mTORC1 and mTORC2. These complexes regulate multiple cellular processes, including cell growth, cell cycle, cell survival, and autophagy. mTORC1 is stimulated by growth factors but inhibited by acid deprivation, hypoxia, energy stress, endoplasmic reticulum stress, genotoxic stress, and AMPK activity. mTORC2 is relatively insensitive to nutritional deficiencies (Yuan J. et al., 2019). mTOR signaling is closely related to the aging processes in various organisms such as yeasts, worms, flies, and mammals (Lamming et al., 2013; Zheng et al., 2021). mTOR is also a key inhibitor of autophagy, and inhibition of the mTOR signaling pathway promotes autophagy. Enhanced autophagy is not only associated with the aging processes but also implicated in several aging-related pathologies, including cancer, metabolic diseases, and neurodegenerative diseases (Johnson et al., 2013).

## 4 Conclusion, limitations, and perspectives

Aging is a complex physiological process influenced by various factors, particularly environmental and genetic factors. The understanding of mechanisms underlying aging has evolved from intuitive perception to molecular exploration over the past decades, with investigation of the most prominent genes such as ctl-1, sir-2.1, sod-3, and daf-16 genes. On the other hand, oxidative enzymes have been enhanced to perform antiaging functions, such as enhancing FOXO3a and Akt protein expressions and enhancing SOD and CAT activities in the body. There is a remarkable breakthrough in understanding the biological mechanisms underlying aging, but there is still a long way to go to delay aging and deter aging-related diseases in humans. Chinese medicine and its natural active ingredients have been extensively explored as anti-aging agents recently. They are utilized for their ability to enhance the quality of life of patients and prolong survival. These natural products are effective against aging-related diseases with the presence various bioactive substances. They target the pathogenesis of different diseases, including the elimination of free radicals, inhibition of inflammation, enhancement of multiple oxidative enzyme activities, inducing cell cycle arrest, regulation of oncogenes, prevention of diabetes, inducing adipocyte reduction, and interaction with multiple pathways. Furthermore, nutrition and natural products are particularly popular among older adults, suggesting that dietary natural products are alternative and complementary treatment options that can promote healthy aging and control aging-related adverse effects.

This study provides a broad overview of the use of natural products to treat aging-related diseases and highlights the nutrient-sensing pathways involved in the longevity-extending effects of natural products. The research acknowledges the urgent global health challenge posed by the rising burden of aging-related diseases and the potential benefits of natural products in treating these conditions. One of the major achievements of this study is that it provides a theoretical background for the use of natural products as potential anti-aging agents. The study summarizes the current understanding of nutrient-sensing pathways implicated in the longevity-extending effects of natural products and identifies several recently identified natural products with potential anti-aging properties. This paper also discusses the resources and molecular mechanisms of the active ingredients of natural products that are involved in treating aging-related diseases. Recent studies have shown that many natural products can target key antiaging molecules, such as mTOR, AMPK, SIRT1, and p53. The underlying antiaging mechanisms seem to be resolved at the genetic and molecule levels. For example, in natural product therapeutic pharmacology, rapamycin inhibits mTOR activity and BBR and resveratrol activate AMPK and SIRT1 activities, respectively. It is worth noting that current research on the therapeutic prospects of natural products in the aging process is mainly based on basic laboratory studies. However, the study has limitations, primarily due to the lack of in-depth analysis of individual compounds and their specific effects on aging-related pathways. Another limitation is that the review primarily focuses on cellular and molecular mechanisms and does not address broader issues such as regulatory, economic, and social barriers to using natural products as a viable treatment option for aging-related diseases.

It is worth noting that current research on the therapeutic prospects of natural products in the aging process is mainly based on basic laboratory studies. However, epidemiological studies and clinical trials on dietary and natural product supplements are lacking regarding aging-related issues. The specific antiaging active molecules in certain natural products and the synergism among them are unclear. Furthermore, the effective and safe doses remain to be determined. The major challenge for developing natural products as chemopreventive or antiaging agents are the low contents of effective components and oral bioavailability. Therefore, further research is required to increase the number of large and well-controlled clinical trials determining the safety, efficacy, and tolerability of natural products and understanding the reagent effects of known and unknown antiaging plant compounds. Although a tremendous amount of work still lies ahead, there is no doubt that the discovery of natural products capable of preventing and treating multiple age-related diseases in humans will be valuable. We anticipate that this study will offer theoretical support for the use of current dietary supplements and natural products as promising anti-aging interventions.
